# Molecular Fe(ii)–Ln(iii) dyads for luminescence reading of spin-state equilibria at the molecular level

**DOI:** 10.1039/d4dt01868k

**Published:** 2024-09-18

**Authors:** Timothée Lathion, Neel Deorukhkar, Charlotte Egger, Homayoun Nozary, Claude Piguet

**Affiliations:** a Department of Inorganic and Analytical Chemistry, University of Geneva 30 quai E. Ansermet CH-1211 Geneva 4 Switzerland Claude.Piguet@unige.ch; b CNRS – CBM Rue Charles Sadron CS 80054 45071 Orleans Cedex 2 France

## Abstract

Due to the primogenic effect, the valence shells of divalent iron Fe(ii) ([Ar]3d^6^) and trivalent lanthanides Ln(iii) ([Xe]4f^*n*^) are compact enough to induce spin-state equilibrium for the 3d-block metal and atom-like luminescence for the 4f-block partner in Fe(ii)–Ln(iii) dyads. In the specific case of homoleptic pseudo-octahedral [Fe(ii)N_6_] units, programming spin crossover (SCO) around room temperature at normal pressure requires the design of unsymmetrical didentate five-membered ring chelating N^∩^N′ ligands, in which a five-membered (benz)imidazole heterocycle (N) is connected to a six-membered pyrimidine heterocycle (N′). Benefiting from the *trans* influence, the facial isomer *fac*-[Fe(ii)(N^∩^N′)_3_]^2+^ is suitable for inducing SCO properties at room temperature in solution. Its connection to luminescent [LnN_6_O_3_] chromophores working as non-covalent podates in the triple-stranded [Fe(ii)Ln(L10)_3_]^5+^ helicates (Ln = Nd, Eu) controls the facial arrangement around Fe(ii). The iron-based SCO behaviour of the 3d–4f complex mirrors that programmed in the mononuclear scaffold. Because of the different electronic structures of high-spin and low-spin [Fe(ii)N_6_] units, their associated absorption spectra are different and modulate the luminescence of the appended lanthanide luminophore *via* intramolecular intermetallic energy transfers. It thus becomes possible to detect the spin state of the Fe(ii) center, encoded by an external perturbation (*i.e.* writing), by lanthanide light emission (*i.e.* reading) in a single molecule and without disturbance. Shifting from visible emission (Ln = Eu) to the near-infrared domain (Ln = Nd) further transforms a wavy emitted signal intensity into a linear one, a protocol highly desirable for future applications in data storage and thermometry.

## Abstract 2

Efficient and easy lanthanide(iii)-based luminescence reading of iron(ii) spin-state equilibria presents an attractive challenge, owing to the delicate combination of the primogenic effect and the Fermi golden rule.

## Graphical abstract

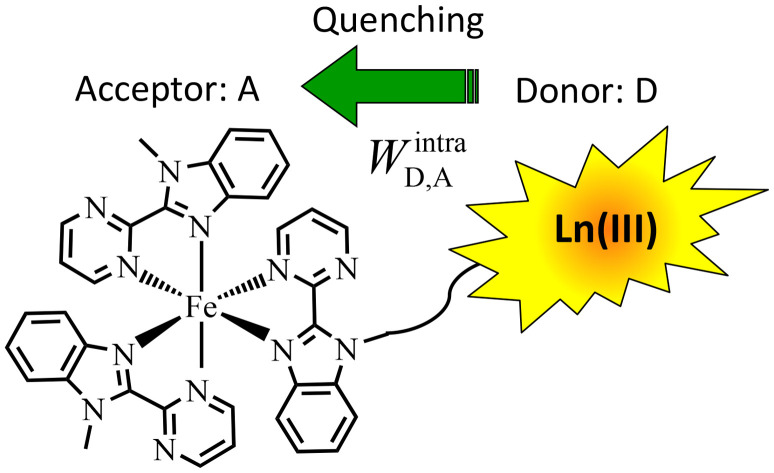

## Introduction

**Fig. 1:**
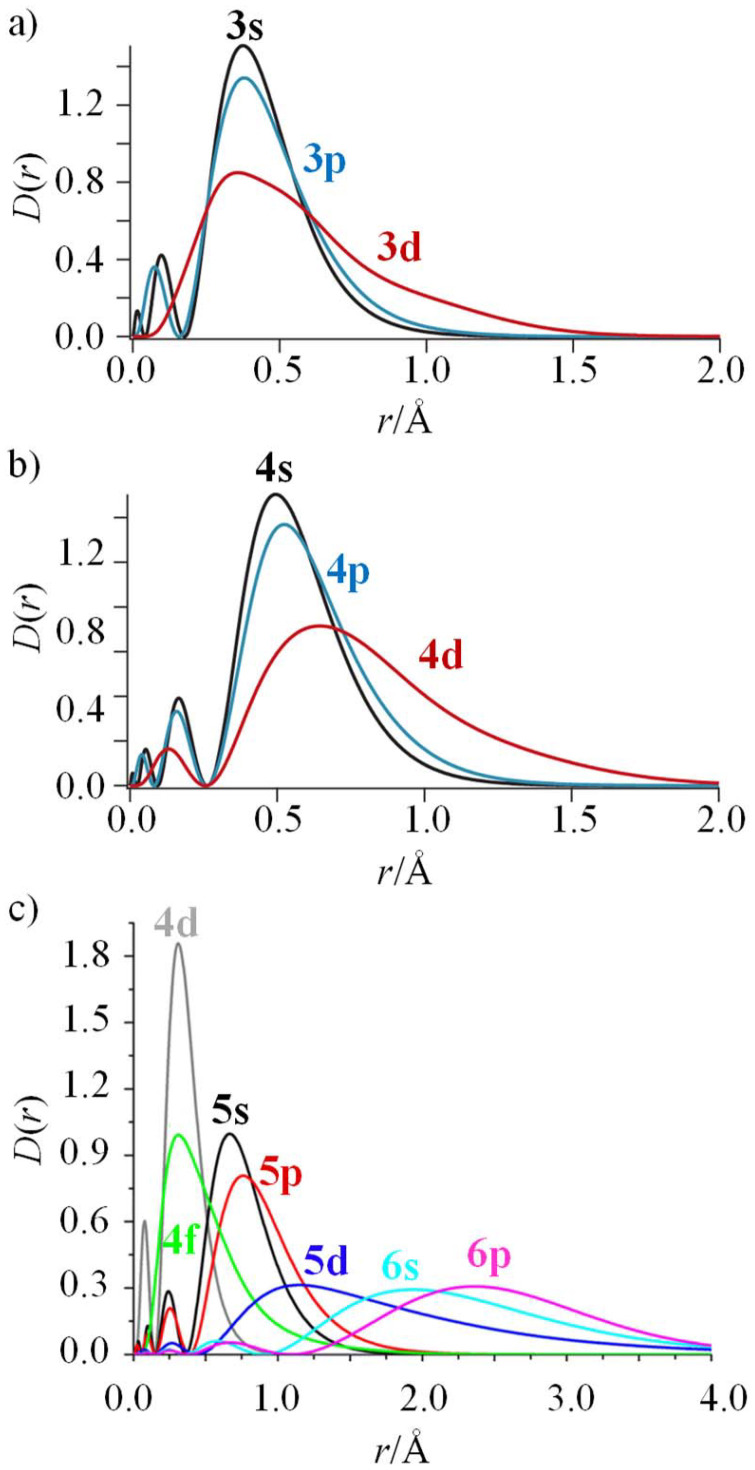
The primogenic effect (lack of a radial node for *r* ≠ 0 for the orbital with *n* − *l* = 1) illustrated by the radial densities *D*(*r*) = *r*^2^*R*(*r*)^2^ for (a) the *n* = 3 shell of Fe(ii),^3^ (b) the *n* = 4 shell of Ru(ii)^3^ and (c) the *n* = 4, 5 shells of Sm(iii).^7^ These figures have been adapted from ref. 3 (a and b), with permission from Science & AAAS, copyright 2019, and ref. 7 (c), with permission of American Chemical Society, copyright 2019.

Due to the lack of radial nodes for distance *r* ≠ 0 in the wave functions of orbitals characterized by *n* − *l* = 1, where *n* and *l* are the principal and azimuthal quantum numbers, respectively, a property referred to as the primogenic effect,^1–3^ the electronic distributions remain compact in 2p, 3d and 4f valence shells,^4–7^ which limits overlap, covalency and perturbation by peripheral atoms (Fig. 1).

**Fig. 2:**
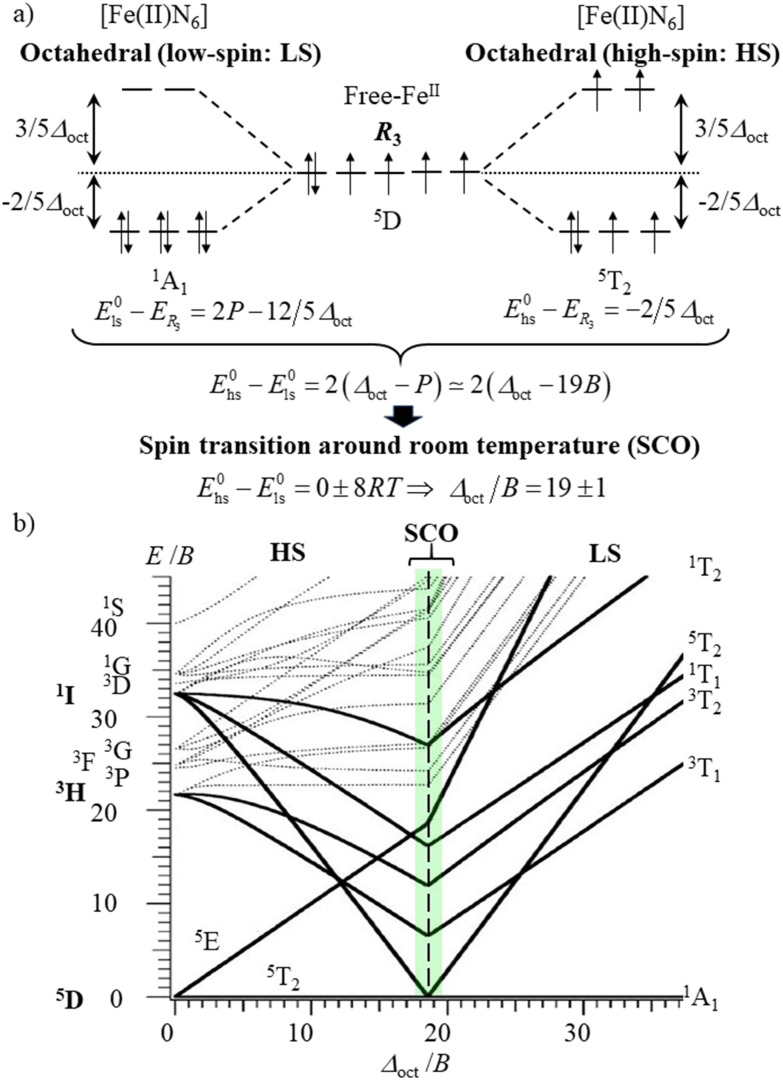
(a) Crystal field/ligand field approach to the spin crossover phenomenon for a d^6^ electronic configuration in an octahedral complex (*P* is the electron spin pairing energy) and (b) Tanabe–Sugano diagram for a d^6^ metal ion.

**Fig. 3:**
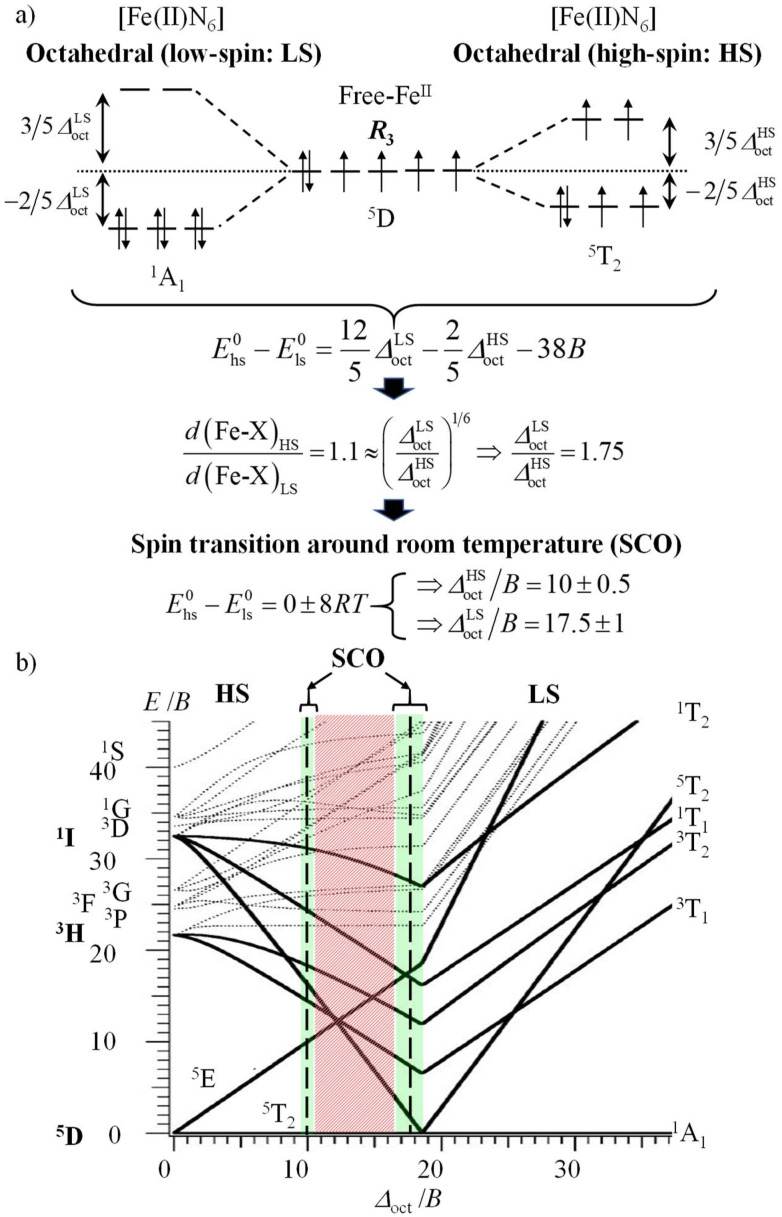
(a) Ligand field approach to the spin crossover phenomenon for a d^6^ electronic configuration in an octahedral complex with specific ligand field strengths and (b) modified Tanabe–Sugano diagram for a d^6^ metal ion.^18–21^

In the case of the complexation of N-donor ligands (nitrogen atoms have compact 2p valence orbitals) to [Ar]3d^*n*^ transition metal ions in coordination chemistry, the primogenic effect limits ligand-field splitting to such an extent that it becomes competitive with spin pairing energies produced by interelectronic repulsions (Fig. 1a). In contrast, the larger expansion characterizing 4d-block systems produces large orbital overlap and ligand fields much larger than spin pairing interactions (Fig. 1b). Consequently, spin-state equilibria become accessible at moderate temperature and/or pressure ranges only for coordination complexes with 3d-block metallic centers.^8^ This phenomenon was recognized by Pauling in the late twenties, but in 1931 he erroneously resorted to hybridization and valence bond theory for tentatively explaining the existence of four unpaired electrons in paramagnetic [Fe(OH_2_)_6_]^2+^ (assigned to electrostatic Fe–O bonds) and no unpaired electron in diamagnetic [Fe(CN)_6_]^4−^ (assigned to covalent Fe–C bonds).^9,10^ He however fully recognized that two molecular systems with different spin states could co-exist at a given temperature *T*, provided that the energy difference between them is comparable with thermal energy (*mRT* with 1 ≤ *m* ≤ 8).^11,12^ The concomitant isolation by Cambi *et al.* of Fe(iii) complexes with dithiocarbamate ligands displaying thermal spin-state equilibria is thus considered as the first experimental demonstration of what is known as the spin crossover (SCO) phenomenon.^13^ Its rationalization had to wait for the development of the ligand field theory during the fifties (Fig. 2a, where *Δ*_oct_ stands for the ligand-field splitting of the d orbitals and *B* is the Racah parameter measuring interelectronic interactions),^14–16^ and its illustration by the Tanabe–Sugano diagrams (Fig. 2b).^17^ Searching for ligands compatible with spin-state equilibria in 3d^6^-Fe(ii) according to the criteria *Δ*_oct_/*B* = 19 ± 1 (Fig. 2) proved difficult, if not impossible, for more than three decades. In 1953, Orgel^18^ first pointed out that the approach leading to Fig. 2b is misleading since the ligand-field splitting is different in the high-spin (HS) and low-spin (LS) electronic configurations due to the presence of electrons in the antibonding e*_g_ orbitals for the HS configuration, which extends the Fe(ii)–X bonds by *circa* 10% (Fig. 3a).^19^ A corrected model (Fig. 3a) then established that ligands compatible with the induction of Fe(ii)-SCO indeed required *Δ*^HS^_oct_/*B* = 10 ± 0.5 or *Δ*^LS^_oct_/*B* = 17.5 ± 1 (green bands in Fig. 3b), whereas the 10.5 ≤ *Δ*_oct_/*B* ≤ 16.5 domain is not accessible to any complex, since it is energetically more favourable for the complex to either contract and form a low-spin complex or to expand and form a high-spin complex (red band in Fig. 3b).^20,21^

With this in mind, it is not so surprising that the first example of an iron(ii) SCO system was reported by Madeja and König only in 1963 for heteroleptic [Fe(phen)_2_X_2_] complexes (X = halides or pseudohalides).^22^ Since then, hundreds (and probably thousands) of homo- and heteroleptic iron(ii) complexes matching the green bands illustrated in Fig. 3b have been synthesized, published and regularly reviewed.^8,19,20,23–31^

Although SCO processes can be induced by pressure, magnetic or electric field, light irradiation and the presence/absence of guest molecules,^32–35^ the most common perturbation is a change in temperature due to its facile application and measurement.^8^ Moreover, it is worth stressing here that the large majority of studies are conducted in the solid state for being able to induce SCO at any accessible temperature and for benefiting from long-range interactions that may result in abrupt spin transitions, cooperativity and hysteresis,^36–38^ properties that are required for the application of SCO materials in information storage.^39–43^ Studies in solution limit SCO processes to the single molecule level and remove cooperative effects, but weak intermolecular interactions with solvent molecules or counterions, or intramolecular communication in multinuclear systems may be exploited for some (ultra) fine tuning of spin-state equilibria.^44–47^ If we now turn our attention toward the trivalent lanthanides and their [Xe]4f^*n*^ electronic configurations, the primogenic effect is more pronounced than with the 3d-block systems, and the maximum of the radial distribution density of the 4f^*n*^ valence shell coincides with that of the filled 4d^10^ orbitals, while the significantly more expanded and filled 5s^2^ and 5p^6^ orbitals protect the 4f electrons from external perturbations (Fig. 1c).^7^ The resulting negligible ligand fields prevent the detection of spin-state equilibria at accessible temperatures and pressures, and additionally, the trivalent 4f-block centers of Ln(iii) retain their atomic electronic properties in their coordination complexes.^48–51^ Combining 3d-block Fe(ii)-SCO units with 4f-block atom-like luminophores, thanks to nitrogen-based segmental ligands, therefore provides switchable Fe(ii)–Ln(iii) dyads, in which the thermal writing of the magnetic information on the iron center can be detected independently through the modulation of the lanthanide luminescence. This strategy seems particularly promising for the design of quantum switches and thermal sensors at the level of a single molecule, where both room temperature SCO and adjustable Ln-based luminescence can be simultaneously programmed.^41,52–54^ The specific Fe(ii)–Ln(iii) communication occurring at the single molecular level considered in this Frontier article is reminiscent of the recent interest in designing solid-state multifunctional magnetic/optical lanthanide-containing materials, where the different outputs can be combined for deciphering the electronic structures and for extending applications in molecular Q-bit design and thermometry. These aspects are regularly reviewed,^55–58^ sometimes with specific focus on d–f interactions,^59–61^ but are not considered further in this contribution which is focused on the specific lanthanide-based luminescence reading of SCO spin-state equilibria occurring in isolated molecules in solution. Similarly, the optical consequences of SCO processes in solid-state magnetic materials are a topic of modern interest particularly in relation to modulating the absorption/emission spectra of neighbouring emissive probes (often polyaromatic ligands).^25,39–43,52,54,62^ Beyond the rare instance of enhanced luminescence reported for a Tb-spin crossover nanocomposite that allows spin state monitoring,^63^ we highlight below what we believe to be the only cases of SCO-modulated Fe(ii)–Ln(iii) communications operating in single molecules in solution.^62^

## Tuning molecular pseudo-octahedral [Fe(ii)N_6_] building blocks for inducing SCO around room temperature

**Fig. 4:**
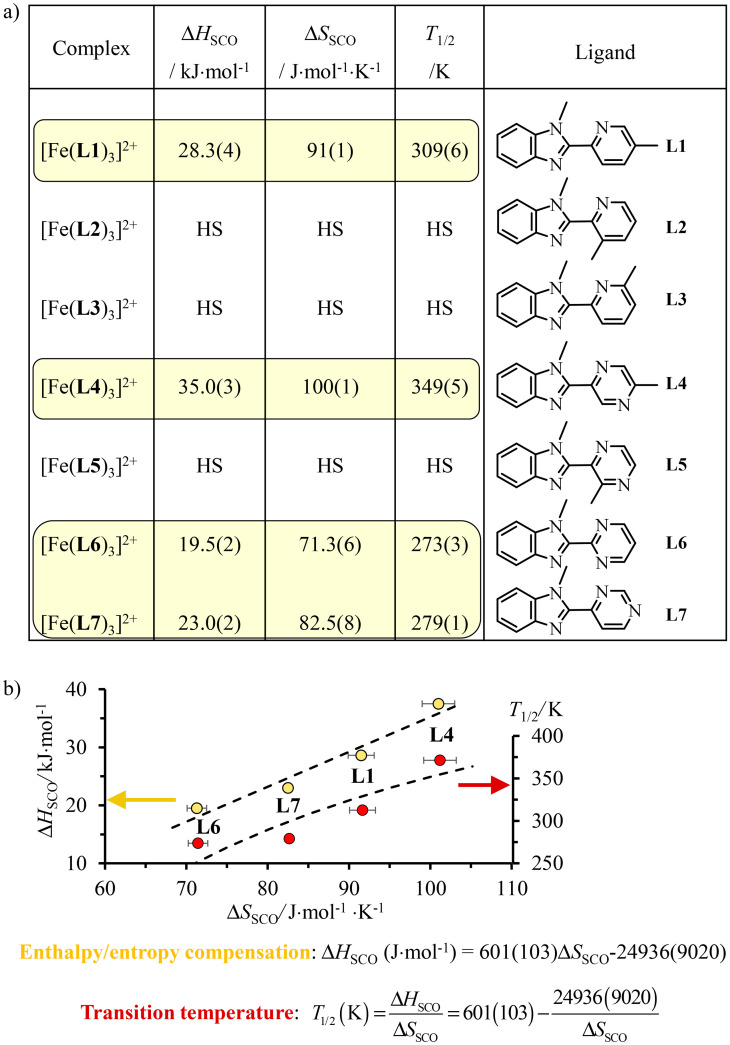
(a) Enthalpies (Δ*H*_SCO_), entropies (Δ*S*_SCO_) and critical transition temperatures *T*_1/2_ = Δ*H*_SCO_/Δ*S*_SCO_ and (b) linear enthalpy/entropy compensation for spin crossover processes operating for [Fe(ii)(L*k*)_3_]^2+^ in acetonitrile.^66–68^

A special approach to the SCO domain from the low-spin side (right part of the green bands, *Δ*^LS^_oct_/*B* = 17.5 ± 1 in Fig. 3b) is well-established for distorted pseudo-octahedral [Fe(ii)N_6_] units, where N stands for heterocyclic nitrogen donors.^19,24,26–29^ For stability reasons, chelate didentate N^∩^N or tridentate N^∩^N^∩^N ligands are preferred over monodentate analogues. Additionally, Shatruk and coworkers recommended the relevant use of unsymmetrical didentate N^∩^N′ ligands with five-membered chelating α,α′-diimine donor groups obtained by the connection of a five-membered aromatic heterocycle (imidazole, pyrazole, benzimidazole, *etc*.) with a six-membered heterocycle (pyridine, pyrazine, pyrimidine, *etc*.) in order to achieve suitable ligand-field strengths for SCO behaviour in [Fe(ii)(N^∩^N′)_3_]^2+^ complexes.^64^ With this in mind, a series of these complexes has been prepared in which a common alkylated benzimidazole group (5-membered heterocycle) is connected to a 6-membered heterocyclic pyridine (L1–L3), pyrazine (L4–L5) or pyrimidine ring (L6–L7) possessing tunable electronic properties (σ-donor/π-acceptor) relevant to SCO^65^ and specific steric constraints (methyl groups, Fig. 4).^66–68^ The pseudo-octahedral [Fe(ii)(N^∩^N′)_3_]^2+^ complexes with N^∩^N′ = L1, L4, L6 and L7 undergo thermally-induced spin crossover (SCO) processes in acetonitrile during which the compact and enthalpically-favored low-spin (LS) diamagnetic ground state (^1^A_1_ in *O*_h_ symmetry) can be switched toward its expanded and entropically favored high-spin (HS) paramagnetic form (^5^T_2_ in *O*_h_ symmetry) according to eqn (1) with Δ*H*_SCO_ > 0 and Δ*S*_SCO_ > 0 (Fig. 4).1
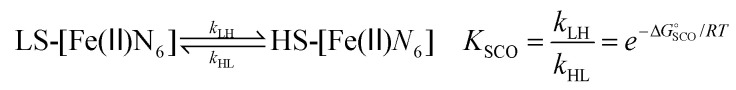


In contrast, [Fe(ii)(L*k*)_3_]^2+^ (L*k* = L2, L3 and L5) remains purely high-spin at all temperatures because steric constraints due to peripheral methyl groups prevent the contraction of the Fe(ii)–N bonds required for adopting the low-spin configuration. As expected for the extension of the Fe(ii)–N bond length accompanying the LS → HS spin transition, the minimum contact distance pertinent to the binding potential is not affected along the ligand series and linear enthalpy/entropy compensation occurs (Fig. 4b, yellow disks).^69,70^ The strong coupling regime, characterized by a negative free energy of compensation of −25(9) kJ mol^−1^, is responsible for an hyperbolic dependency of the critical transition temperature *T*_1/2_ = Δ*H*_SCO_/Δ*S*_SCO_, *i.e.* the temperature at which the LS and HS configurations exist as a 1 : 1 mixture, as a function of the entropy (Fig. 4b, red disks). Consequently, *T*_1/2_ is minimum for the smallest SCO entropy changes and the 2-benzimidazole-pyridimidine ligand L6 seems to be the best candidate to induce SCO behaviour around room temperature (*T*_1/2_ = 279(1) K).^68^

**Fig. 5:**
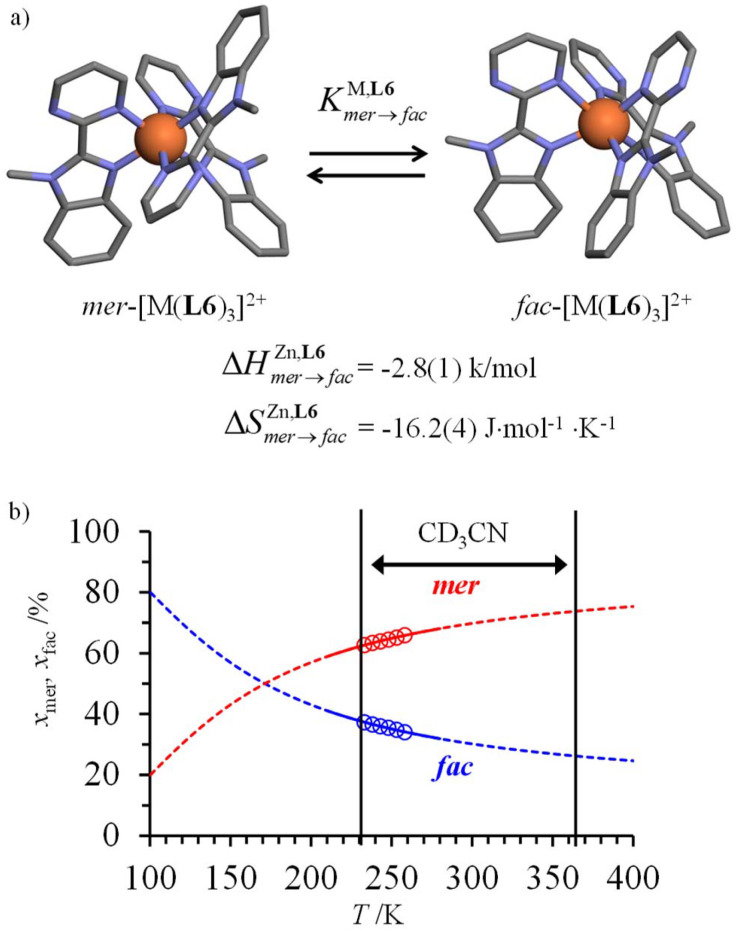
(a) Meridional to facial isomerization of [Fe(ii)(L6)_3_]^2+^ occurring in acetonitrile and (b) associated speciation as a function of temperature (the circles correspond to the experimental data estimated by variable-temperature ^1^H-NMR).^68^

However, one should not underestimate the importance of meridional/facial isomerism for [Fe(ii)(N^∩^N′)_3_]^2+^ complexes exhibiting the SCO processes,^29^ particularly for data recorded in solution where no long-range intermolecular constraints are present and both isomers co-exist in variable and non-negligible amounts (Fig. 5a).

**Fig. 6:**
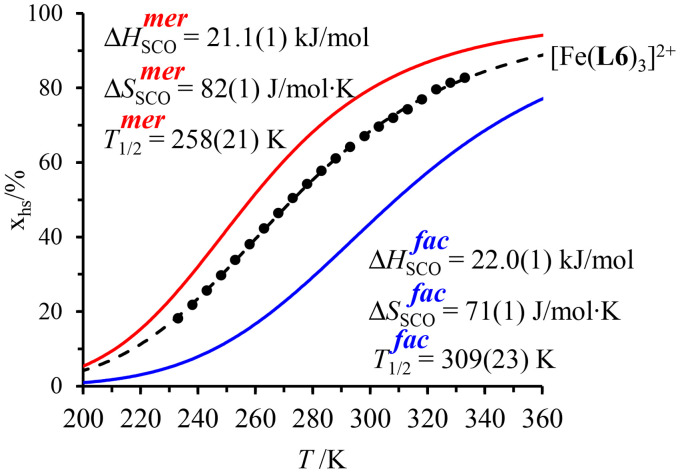
Mole fractions of HS-[Fe(ii)(L6)_3_]^2+^ for meridional and facial isomers in acetonitrile as a function of temperature during the spin transition.^68^

In this context, the variable-temperature ^1^H-NMR speciation of the diamagnetic [Zn(L6)_3_]^2+^ model complex in acetonitrile (Fig. 5b) gave linear van't Hoff plots from which the thermodynamic characteristics of the isomerization equilibrium became accessible.^68^ The slightly negative enthalpy 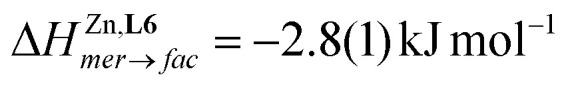 observed for the *mer* → *fac* isomerization points to a stabilization of the latter isomer due to the thermodynamic *trans* influence.^71^ The opposite positive entropic contribution 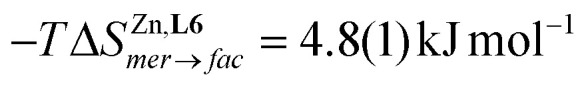 at room temperature, which combines the statistical gain in degrees of freedom for the meridional isomer with some unfavorable organization of the second sphere solvent molecules around the facial isomer,^71^ stabilizes the alternative meridional isomer. Reasonably assuming that the *mer*/*fac* speciation measured for [Zn(L6)_3_]^2+^ also holds for [Fe(ii)(L6)_3_]^2+^ under the same conditions, the analysis of the magnetic susceptibility curves as a function of temperature provides the mole fraction of HS-[Fe(ii)(L6)_3_]^2+^ (black trace in Fig. 6), which can be split into two contributions specifically assigned to *mer*-[Fe(ii)(L6)_3_]^2+^ (red trace in Fig. 6) and *fac*-[Fe(ii)(L6)_3_]^2+^ (blue trace in Fig. 6), respectively.^68^ As expected from the stabilizing *trans* influence that strengthens the Fe(ii)–N bonds in *fac*-[Fe(ii)(L6)_3_]^2+^, Δ*H*^*fac*^_SCO_ > Δ*H*^*mer*^_SCO_ and the critical transition temperatures *T*_1/2_ = Δ*H*_SCO_/Δ*S*_SCO_ of the two isomers diverge, a trend further boosted by Δ*S*^*fac*^_SCO_ > Δ*S*^*mer*^_SCO_ (Fig. 6). Altogether, *T*^*mer*^_1/2_ = 258(21) K and *T*^*fac*^_1/2_ = 309(12) K make *fac*-[Fe(ii)(L6)_3_]^2+^ the best candidate for inducing SCO around room temperature.

## Lanthanide-based luminescence detection of Fe(ii) SCO in single (supra)molecules

**Fig. 7:**
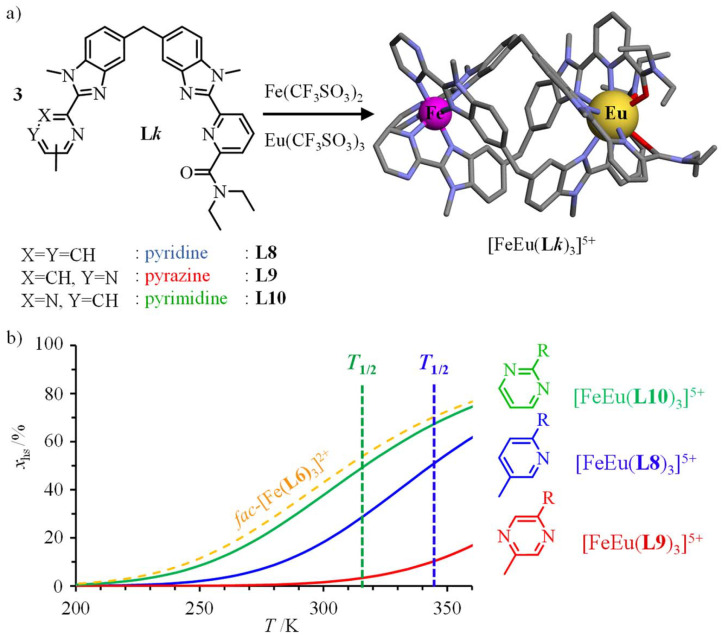
(a) Self-assembly of triple-stranded [Fe(ii)Eu(L*k*)_3_]^5+^ helicates in acetonitrile and (b) associated SCO properties showing the mole fractions of HS-[Fe(ii)Eu(L*k*)_3_]^5+^ as a function of temperature.^66,75,76^ The SCO curve for *fac*-[Fe(ii)(L6)_3_]^2+^ (dashed orange trace) has been added for comparison purposes.

Helicate self-assembly^72,73^ appears to be well-suited for connecting *fac*-[Fe(ii)(L*k*)_3_]^2+^ (L*k* = L1, L4, L6) building blocks to luminescent nine-coordinated emissive trivalent lanthanide cations working as a non-covalent tripod in [Fe(ii)Ln(L*k*)_3_]^5+^ (L*k* = L8–L10, Ln = Nd, Eu; Fig. 7a).^66,74–76^ As expected from the data collected for mononuclear models, the associated [Fe(ii)Eu(L*k*)_3_]^5+^ triple-stranded helicates exhibit SCO in solution with critical transition temperatures stepwise decreasing in the order *T*_1/2_([Fe(ii)Eu(L9)_3_]^5+^ = 412(8) K, pyrazine-benzimidazole) > *T*_1/2_([Fe(ii)Eu(L8)_3_]^5+^ = 344(3) K, pyridine-benzimidazole) > *T*_1/2_([Fe(ii)Eu(L10)_3_]^5+^ = 317(1) K, pyrimidine-benzimidazole), the latter heterometallic complex being unique for approaching room temperature SCO (Fig. 7b). Moreover, the spin transition in [Fe(ii)Eu(L10)_3_]^5+^ almost exactly fits that found in the mononuclear *fac*-[Fe(ii)(L6)_3_]^2+^ model (compare green and orange traces in Fig. 7b).

**Fig. 8:**
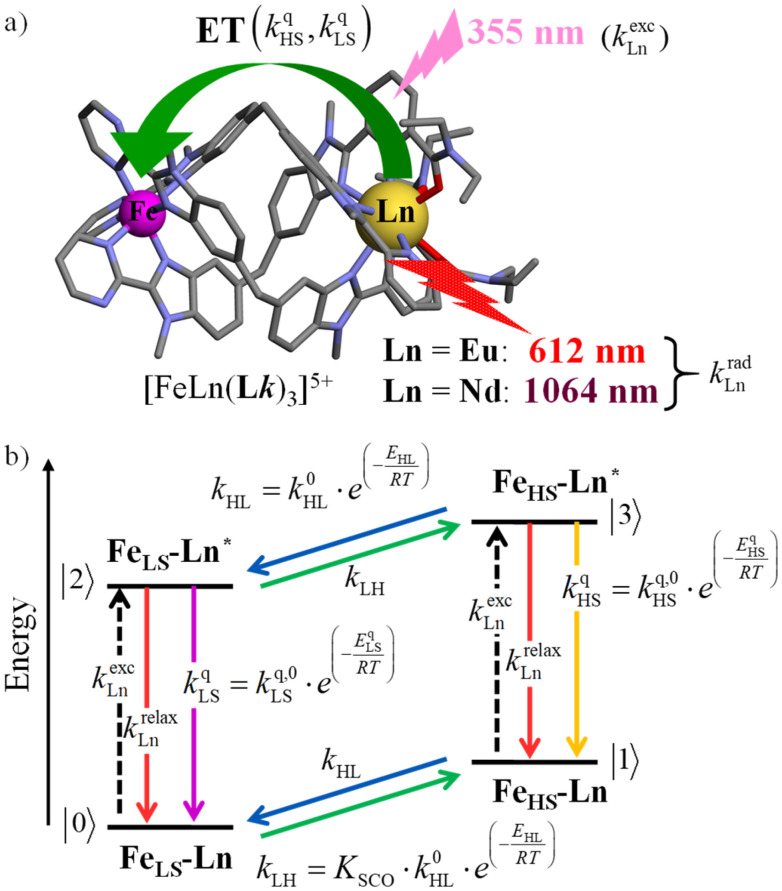
(a) Fe(ii)-modulated light downshifting operating in [Fe(ii)Ln(L10)_3_]^5+^ helicates (ET = energy transfer) and (b) associated four-states kinetic model.

The choice of Ln(iii) along the 4f block series has only minor influence on the SCO properties of the appended [Fe(ii)N_6_] unit, but the resulting luminescence drastically depends on the selected lanthanide. The Ln(iii)-based light emission process can be highlighted by a simple kinetic model, which considers simultaneously the Fe(ii)-based SCO process and its influence on the photophysical properties of the lanthanide emitter in [Fe(ii)Ln(L10)_3_]^5+^ (Fig. 8). Beyond the well-known radiative (*k*^rad^_Ln_), responsible for luminescence with a maximum at 612 nm for Ln = Eu and at 1064 nm for Ln = Nd, and non-radiative (mainly of vibrational origin: *k*^non-rad^_Ln_) contributions to the global relaxation process (*k*^relax^_Ln_ = *k*^rad^_Ln_ + *k*^non-rad^_Ln_), the presence of the appended SCO Fe(ii) center provides two additional quenching pathways *via* Ln → Fe(ii) energy transfers toward the low-spin (*k*^q^_LS_) or high-spin (*k*^q^_HS_) states, respectively (Fig. 8).^75–77^

In the absence of intermetallic energy transfers (*k*^q^_LS_ = 0 and *k*^q^_HS_ = 0), for instance, when Fe(ii) is replaced with closed-shell Zn(ii) in [ZnLn(L10)_3_]^5+^, the Ln-based luminescence is strictly controlled by *k*^relax^_Ln_ = *k*^rad^_Ln_ + *k*^non-rad^_Ln_, and one can roughly predict (and observe for Ln = Eu)^76^ a simple decrease of the steady-state luminescence intensity with increasing temperature. In [Fe(ii)Ln(L10)_3_]^5+^, the relaxation of the excited Ln* state is further affected by the two specific Ln → Fe(ii) energy transfers (*k*^q^_LS_ ≠ *k*^q^_HS_ ≠ 0), given that the distribution of each spin state is temperature-dependent *via x*_HS_/*x*_LS_ = *k*_LH_/*k*_HL_ = *K*_SCO_ = exp(Δ*S*_SCO_/*R* − Δ*H*_SCO_/*RT*). Focusing on the energy transfer theory, the intermetallic communication obeys the Fermi golden rule (eqn (2)), where *W*^intra^_DA_ is the rate constant for the resonant energy transfer from the donor (Ln(iii)) toward the acceptor (LS-Fe(ii) or HS-Fe(ii)), 
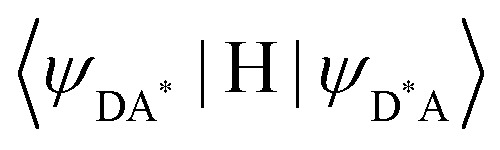
 is the coupling between the two transition multipoles, expressed for a molecular D–A pair (*H* is the interaction Hamiltonian that mediates energy transfer from the excited donor D* to the ground-state acceptor A) and 
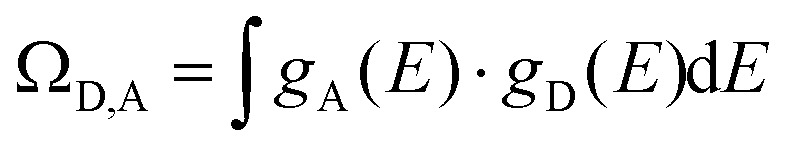
 is the spectral overlap integral ensuring energy conservation, with *g*_D_(*E*) and *g*_A_(*E*) being the normalized line shape functions for the homogeneous lines of the donor (Ln(iii)-based emission spectrum) and acceptor (Fe(ii)-absorption spectrum), respectively.^78^2
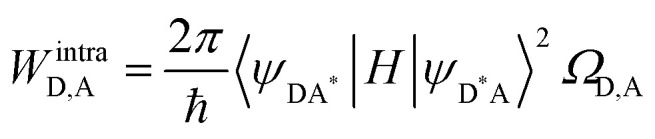


**Fig. 9:**
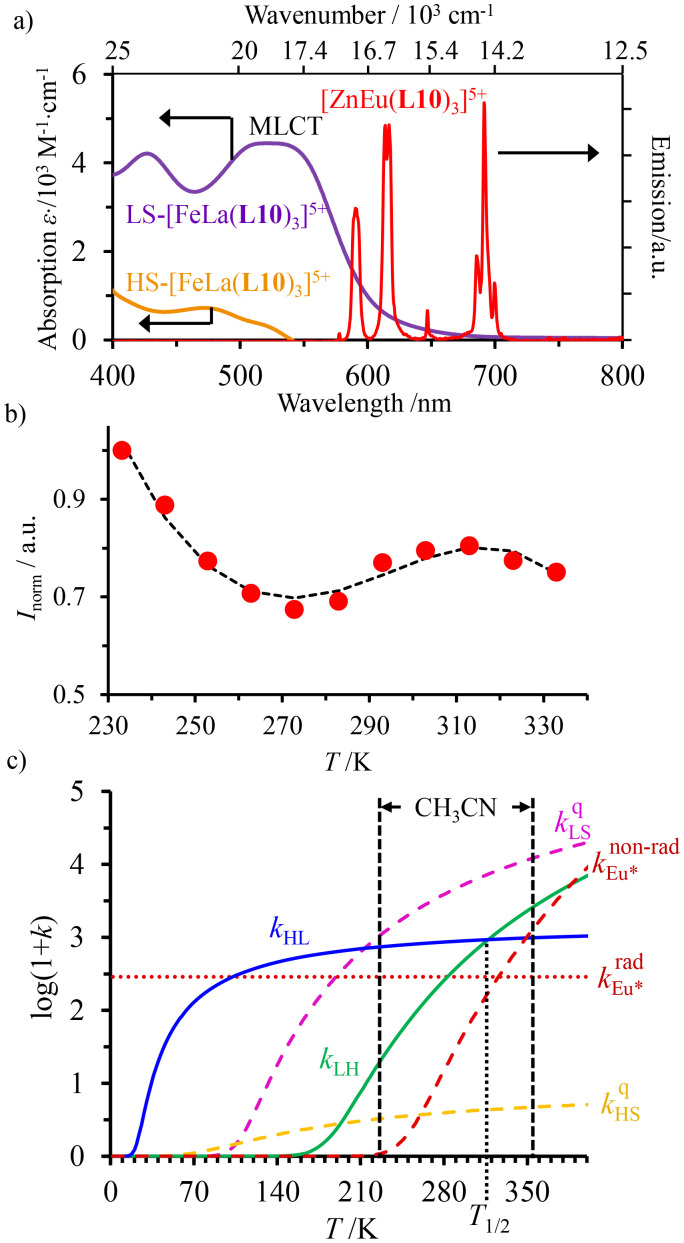
(a) Room temperature electronic absorption spectra recorded for [Fe(ii)N_6_] chromophores in [Fe(ii)La(L10)_3_]^5+^ (low spin: purple trace, high-spin: orange trace) and visible emission spectrum recorded for Eu(iii) in [ZnEu(L10)_3_]^5+^ (red trace) in acetonitrile,^76^ (b) Experimental (red disks) and fitted (black dashed traces) normalized total integrated intensity (*I*/*I*_max_ = *I*_T_/*I*_233 K_) for the emission of Eu(iii) (red disks) in [Fe(ii)Eu(L10)_3_]^5+^ (*λ*_exc_ = 333 nm)^76^ and (c) associated rate constants obtained by fitting the experimental data with the kinetic model shown in Fig. 8b for luminescence monitoring of the Fe(ii) spin-state using Ln(iii) = Eu(iii) 
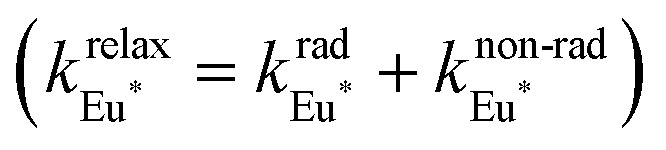
 in acetonitrile. This figure has been adapted from ref. 76 with permission from American Chemical Society, copyright 2024.

Having the absorption spectra of LS-[Fe(ii)N_6_] (purple trace in Fig. 9a) and HS-[Fe(ii)N_6_] (orange trace in Fig. 9a) at hand, it is easy to program *Ω*_D,A_ ≠ 0 when one considers the emission spectrum of Eu(iii) (red trace in Fig. 9a). Moreover, it is anticipated that the energy matching conditions, as estimated by the spectral overlap integral *Ω*_D,A_, will contribute to set *k*^q^_LS_ > *k*^q^_HS_ in [Fe(ii)Eu(L10)_3_]^5+^ and to induce some complicated variations in the luminescence with increasing temperature since strongly quenching LS-[Fe(ii)N_6_] is stepwise transformed into weakly quenching HS-[Fe(ii)N_6_] (Fig. 9b). The kinetic model shown in Fig. 8b provides the steady-state emission intensities from the excited level of the lanthanide (Ln*) as summarized in eqn (3), from which various rate constants can be estimated by non-linear least-squares fits of the experimental normalized emissions recorded as a function of temperature (Fig. 9c; 
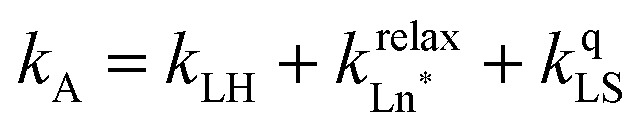
 and 
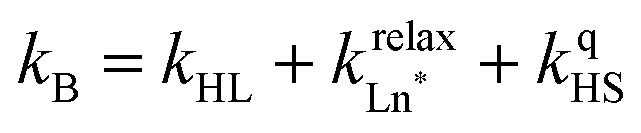
).^77^3
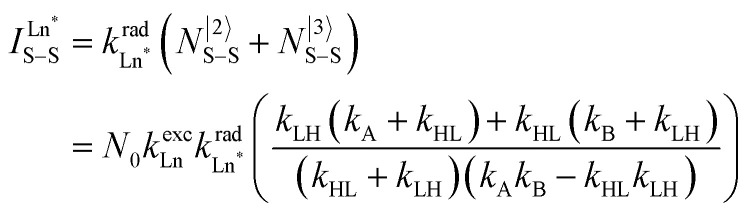


The recalculated Eu-based intensities (dashed black trace in Fig. 9b) fairly reproduce the experimental data (red disks in Fig. 9b). As expected, *k*^q^_LS_ ≫ *k*^q^_HS_ at all temperatures (Fig. 9c), and the wavy shape of the emission curve in solution (230–330 K, Fig. 9b) can be easily explained by the opposite contributions of (i) the vibrational relaxation pathway 
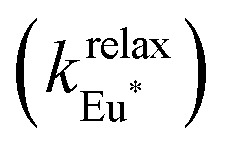
, which increases with increasing temperature and becomes dominant in the 230–270 K range, and (ii) the total Fe(ii)-based quenching *via* energy transfer, which decreases when LS-[Fe(ii)Eu(L10)_3_]^5+^ is converted into HS-[Fe(ii)Eu(L10)_3_]^5+^ and becomes dominant in the 270–320 K range (Fig. 9b). One concludes that, for [Fe(ii)Eu(L10)_3_]^5+^, the wavy modulation of the emission intensity provides the required information for reading the appended Fe(ii) spin state, but only in an indirect way since the concomitant vibrational relaxation processes must also be calibrated.

The ultimate Holy Grail in this writing/reading process should be a linear dependence between the amount of HS-[Fe(ii)N_6_] and the Ln(iii)-based emission. The key to this problem involves constant vibrational relaxation within the 230–330 K range, along with sufficiently differentiated intermetallic Ln → HS-Fe(ii) and Ln → LS-Fe(ii) energy transfers modulating the lanthanide-based luminescence. The first condition can be easily met by replacing the visible Eu(iii) emitter with Nd(iii), which is known to emit in the near-infrared domain (maximum at 1064 nm) with a constant vibrational relaxation in the 230–330 K domain due the small energy gap with respect to the ground spectroscopic level in [Fe(ii)Nd(L10)_3_]^5+^.^77,79^ Focusing on the intramolecular intermetallic energy transfers in [Fe(ii)Ln(L10)_3_]^5+^, one can reasonably consider the operation of only through-space electric dipole/dipole interactions^80,81^ according to eqn (4),^52,82^ where *κ*^2^ is an orientation factor, *N*_A_ is the Avogadro constant in mmol^−1^, *η* is the refractive index of the medium, 
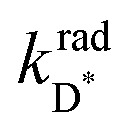
 is the radiative rate constant of the donor and *J*_F_ is the normalized Förster spectral overlap integral in the wavenumber scale.^83^4
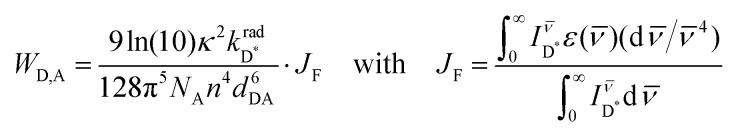


**Fig. 10:**
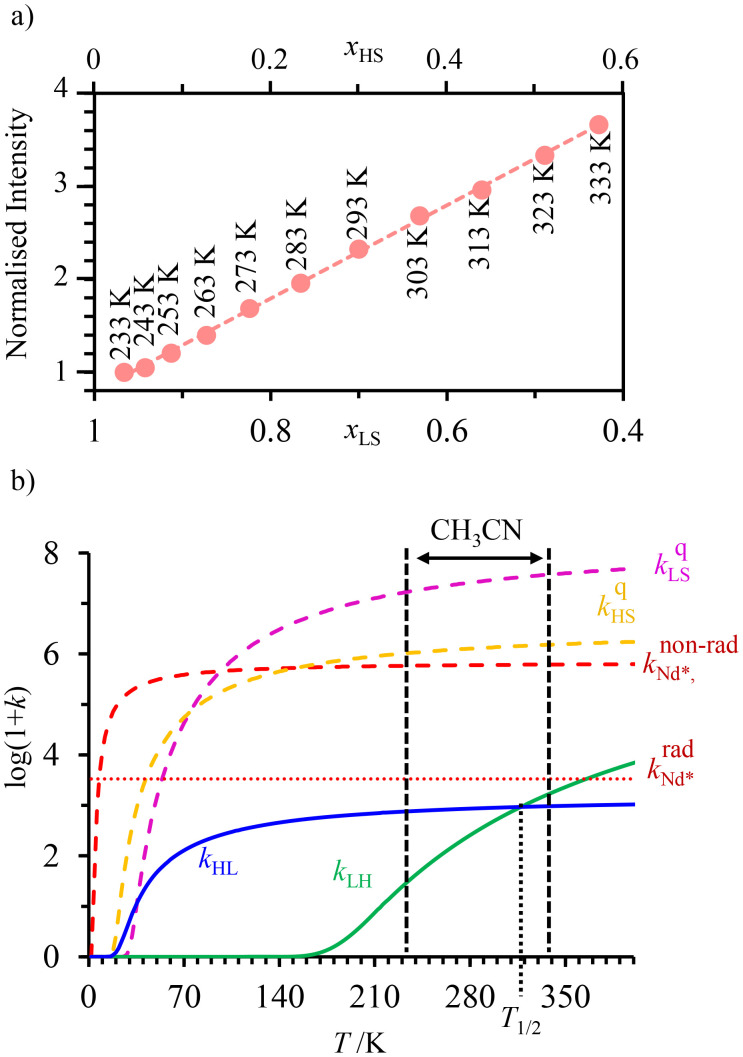
(a) Steady-state Nd(^4^F_3/2_) NIR luminescence monitoring of the Fe(ii) spin-state in the dinuclear [Fe(ii)Nd(L10)_3_]^5+^ helicate in acetonitrile (*x*_LS_ = 1 − *x*_HS_ is the mole fraction of low-spin Fe(ii)) and (b) associated kinetic rate constants (see Fig. 8 for the kinetic scheme). This figure has been adapted from ref. 77 with permission from American Chemical Society, copyright 2024.

One immediately notices that *W*_D,A_ is maximum, and thus pertinent to the efficient and versatile tuning of the residual emission of a Ln(iii) sensitizer acting as an energy donor in [Fe(ii)Ln(L10)_3_]^5+^, when the latter complex possesses a large lanthanide radiative rate constant 
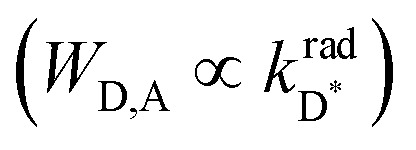
 and a considerable spectral overlap integral 
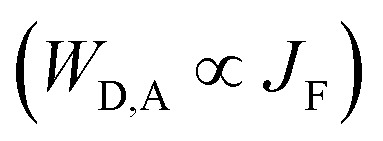
 at low energy 
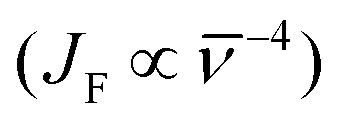
. In this context, replacing Eu(iii) with Nd(iii) to give [Fe(ii)Nd(L10)_3_]^5+^ seems attractive because (i) the visible Eu(^5^D_0_ → ^7^F_*J*_) multiple emission is replaced by the lower energy near-infrared (NIR) Nd(^4^F_3/2_ → ^4^I_*J*_) emission, (ii) the radiative constant of the donor 
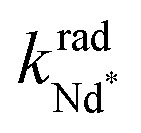
 is increased by one order of magnitude because the involved intrashell Nd(^4^F_3/2_ → ^4^I_*J*_) emission transitions are spin-allowed and (iii) the low-energy spectral overlap between the Nd-based emission spectrum and the Fe(ii)(^5^E ← ^5^T_2_) absorption of HS-[Fe(ii)N_6_] is largely improved.^77^ The detection of the temperature-dependent NIR Nd(iii)-based emission (800–1400 nm) in [Fe(ii)Nd(L10)_3_]^5+^ indeed showed the long-awaited linear correlation with the mole fraction of HS-[Fe(ii)N_6_] unit (Fig. 10a), which could be rationalized by the kinetic model shown in Fig. 8 and the set of rate constants presented in Fig. 10b.

## Conclusions

Thanks to the pertinent analysis of the Tanabe–Sugano diagram established for [Ar]3d^6^ (Fig. 3), efficient tuning of the ligand field strength (*Δ*_oct_) and nephelauxetic effect (*B*) allows a stepwise approach to the SCO domain in pseudo-octahedral [Fe(ii)N_6_]^2+^ units. Stable-room-temperature Fe(ii) spin-state equilibrium in solution requires the design of unsymmetrical didentate five-membered ring chelating ligands to give [Fe(ii)(N^∩^N′)_3_]^2+^, where N and N′ are the nitrogen atoms belonging to a five-membered heterocycle (N) connected to a six-membered heterocycle (N′). Focusing on the most promising candidate 1-methyl-2-(pyrimidin-2-yl)-1*H*-benzo[*d*]imidazole (L6) further requires a rational control of the isomerization processes since only *fac*-[Fe(ii)(L6)_3_]^2+^ is adapted for acting as a building block exhibiting room temperature SCO processes in isolated molecular complexes in solution. With this in mind, one finally takes advantage of thermodynamic self-assembly processes for the quantitative connection of optimized *fac*-[Fe(ii)(N^∩^N′)_3_]^2+^, the organization of which is ensured by an appended luminescent non-covalent [LnN_6_O_3_] tripod in the target [Fe(ii)Nd(L10)_3_]^5+^ triple-stranded helicate. The intermetallic distance of *circa* 1 nm being compatible with intramolecular multipolar through-space energy transfers, the Ln(iii)-based luminescence intensity can be modulated by the appended Fe(ii) spin state since the spectral overlap integral is larger with LS-Fe(ii) acceptors. Although not recognized at first sight, the authors finally realized that near-infrared Nd(iii)-based emission provides the most attractive way to optically detect molecular spin-state equilibria, since a linear correlation exists between the spin state and luminescence, which paves the way to the unambiguous luminescence detection of the SCO process. If molecular switches have to be developed, the requirement of SCO-cooperativity associated with multinuclearity at the (supra)molecular level has to be considered seriously. Until now, polymetallic Fe(ii)-SCO architectures (helicates,^28^ grids^41^ and cages^29,84–86^) had shown only limited deviations from the regular solution model, and substantial efforts are needed along these lines. Moreover, the possibility of inducing double luminescence detection with two different lanthanide ions in the same entity with specific responses remains a crucial challenge if ratiometric reading is to be implemented at the molecular level.^87^

## Data availability

Being a Frontier article, there is neither ESI nor new data.

## Conflicts of interest

There are no conflicts to declare.
